# Challenging Obstetrical Management in Generalized Peritonitis during Pregnancy

**DOI:** 10.1155/2022/1249676

**Published:** 2022-04-21

**Authors:** Susana Patrícia Lima Oliveira, Ana Isabel Sousa, Nuno Nogueira Martins

**Affiliations:** Department of Obstetrics and Gynecology, Centro Hospitalar Tondela-Viseu, Viseu, Portugal

## Abstract

Acute abdomen in pregnancy represents a diagnostic and therapeutic challenge, despite the current advances in modern medicine, since the typical symptoms and altered laboratory parameters mimic normal pregnancy. Acute appendicitis is the most common nonobstetric surgical emergency during pregnancy, with an incidence of 1 per 500-2000 pregnancies. Delayed diagnosis and reluctance to operate on a pregnant woman predispose to adverse maternal and fetal outcomes. The elective termination of pregnancy or interventions to prolong it in the presence of appendicitis is controversial. We present a case of a 38-year-old Caucasian woman, G2P0, admitted to the Obstetric Emergency Department at 13 4/7 weeks of gestation with a primary complaint of severe nausea and vomiting associated with progressive diffuse abdominal pain which had started 7 days before. After the difficulty of inherent differential diagnosis, she was diagnosed with generalized peritonitis due to acute perforated appendicitis. Prompt exploratory laparotomy with appendectomy and drainage of multiple abscesses were performed. Conservative obstetrical management was assumed, with subsequent periodic monitoring of the fetal focus. Due to abdominal compartment syndrome, the abdomen was left open for 4 days. After 7 days in the intensive care unit, recovery was favorable, pregnancy remained uneventful, and a healthy full-term baby was born 27 weeks later. This case represents a successful example of how the cooperation of the obstetrics and general surgery teams and the decision of conservative obstetrical management in the surgical environment contributed to optimizing maternal health, achieving the best obstetrical outcome.

## 1. Introduction

Acute abdomen in pregnancy represents one of the most confronting diagnostic and therapeutic contemporaneous dilemmas, challenging modern medicine [1, 2] . Appendicitis occurs in approximately 1 out of every 500 to 2000 gestations, most commonly seen in the second trimester [3, 4]. It is the main cause of nonobstetrical emergency requiring surgery in pregnancy, representing 25% of all causes [2, 5]. Overall, the prevalence of appendicitis is the same in pregnant and nonpregnant patients [3, 6].

Reaching the correct diagnosis could be laborious due to physiologically anatomical and biochemical adjustments occurring during pregnancy [7]. The classic symptoms—anorexia, nausea, and vomiting—may easily be mistaken for hyperemesis *gravidarum*; pain in the right lower quadrant could be assumed as round ligament pain; and mild leukocytosis is a common laboratory finding in normal gestation [8, 9]. Even though the appendix ascends in the abdominal cavity as the uterus enlarges modifying the location of symptoms, pain in the right lower quadrant of the abdomen is the most common presenting complaint regardless of gestational age [3, 10, 11].

Delays in diagnosis and reluctance to perform surgery on pregnant women confer a greater risk of developing appendicular perforation and subsequent peritonitis, which may lead to other complications including sepsis, abortion, premature labor, and maternal mortality [1, 2, 12].

Unruptured appendicitis is associated with a fetal loss rate of 2% compared to more than 30% in pregnant women with a perforated appendix [13]. Sepsis in pregnancy represents one of the leading causes of death in the intensive care setting, with maternal mortality ranging from 1.8 to 11% and fetal mortality from 10 to 40% depending on gestational age [14].

A high index of suspicion and a low threshold to decide for surgery remains paramount in the management of this potentially life-threatening condition [11, 15]. Evidence does not support that surgery during pregnancy results in an increased incidence of congenital abnormalities [16].

Multidisciplinary cooperation between surgical and obstetrics teams is the cornerstone in ensuring optimal care for the pregnant and the fetus [7]. Clinical decision-making regarding termination or proceeding pregnancy in a critical illness is a controversial issue with arguments on both sides [17, 18].

A case of septic shock in the context of the acute abdomen during pregnancy with successful step-by-step coordination of obstetrics and general surgery teams achieving the best obstetrical outcome despite the difficulty in diagnosis, management, and decision is presented below.

## 2. Case Presentation

A 38-year-old Caucasian woman, G2P0, presented to the Obstetric Emergency Department at 13 4/7 weeks of gestation with a primary complaint of severe nausea and vomiting associated with progressive diffuse abdominal pain which had started 7 days before. She had a history of medicated and clinically controlled bipolar disorder and hypothyroidism and had no relevant family history. She had been to the Obstetric Emergency Department twice before with similar but milder complaints and was discharged with the diagnosis of hyperemesis *gravidarum* and clinical improvement under antiemetic drugs. At readmission, the patient referred progression of emesis to complete oral intolerance and worsening pain in the right lower quadrant in the absence of diarrhea, constipation, dysuria, or anomalous vaginal discharge or bleeding.

She was admitted to the obstetrical observation room, and her vital signs upon arrival were a tympanic temperature of 37.5°C, a blood pressure of 133/81 mmHg, a heart rate of 124 bpm, a respiratory rate of 19 breaths/min, and a 0_2_ saturation of 98% on room air. Generalized muscle guarding was observed on abdominal examination along with a positive *Blumberg* sign.

Obstetrical ultrasound revealed an intrauterine gestational sac containing a fetus with a crown-rump length compatible with 13 4/7 weeks and fetal heart rate (FHR) was 159 bpm. Laboratory findings reported a white blood count of 9.9 × 10^9^/L, and increased values of C-reactive protein (30.94 mg/dL), procalcitonin (144.30 ng/mL), and creatinine (1.6 mg/dL). Arterial blood sample results revealed hyperlactatemia (5.8 mmol/L), hypocalcemia (2 mEq/L), and hyperglycemia (320 mg/dL). The renal ultrasound was normal. Abdominal ultrasound described a moderate amount of peritoneal fluid dispersed into the abdominal cavity, including perihepatic and perisplenic recesses, not referring to the appendix.

Prompt collaboration of the General Surgery Department was requested, and the diagnosis of septic shock in relation to an acute abdomen was confirmed. At that time, blood pressure became immeasurable, and the patient was immediately taken to the operating room for exploratory laparotomy. Under general anesthesia, through a midline incision along the full length of the abdomen, intraoperative findings included a multiperforated and gangrenous appendix and abundant purulent ascites. Multiple abscesses were drained, and after the main surgical procedure, the abdomen was irrigated with copious isotonic fluid.

**Figure 1:**
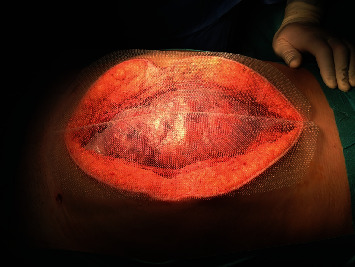
Confection of temporary abdominal closure due to abdominal compartment syndrome.

At that moment, the obstetrics team was confronted with the challenging decision of termination or continuation of the pregnancy. Conservative obstetrical management was assumed, with subsequent periodic monitoring of the fetal focus. Due to severe peritonitis with massive bowel distension and presumed abdominal compartment syndrome, the abdomen was left open and a temporary closure with transparent adherent film and gauze was made (Figure 1).

In the immediate postoperative period, the patient was transferred to the intensive care unit (ICU). Intravenous fluid resuscitation, vasopressors, combined antibiotics, and sedatives were administered. Mechanical ventilation and hemodialysis were required. An intravenous piperacillin/tazobactam antibiotic regimen was used, started on postoperative day 0, and replaced by amoxicillin/clavulanate on postoperative day 6 after microbiological results of the peritoneal fluid collected during surgery that identified *Escherichia coli, Streptococcus mitis, Streptococcus oralis*, and *Streptococcus constellatus ssp constellatus*, all multisensitive. Antibiotic therapy was continued until postoperative day 14.

A first laparostomy revision was performed on postoperative day 2, with mild infection signs and significant bowel edema again preventing abdominal closure. Laparostomy was reconstructed with a polypropylene mesh and delayed primary fascial closure was achieved on postoperative day 4.

During the stay in the ICU, daily FHR was obtained at the bedside by the obstetrics team, assuring fetal vitality. The patient became progressively less dependent on supportive therapy, with no fever records since postoperative day 5 and extubated on day 7. Complete recovery occurred, and she was discharged home on postoperative day 15. No more adverse or unanticipated events occurred.

Pregnancy was then uneventfully carried to full term, with referral to periodic prenatal visits at the hospital. The labor was induced with prostaglandin E2 at 41 0/7 weeks. Due to a nonreassuring fetal status (prolonged deceleration), an emergent cesarean section with midline vertical suprapubic incision and hysterotomy with low-transverse incision was performed. A male weighing 3150 g with an Apgar score of 5/8/10 was born. He was admitted to the Special Newborn Care Unit with a diagnosis of sepsis, but evolution under antibiotic therapy, double intravenous antibiotic therapy with ampicillin and gentamicin from day 1 to day 11 of life, revealed favorable. Vaginal or placental swabs were not collected after the cesarean section because there was no evidence before delivery or during delivery of an infectious condition. The placenta was submitted for histopathological analysis revealing mild vasculitis and funiculitis phenomena. The blood culture of the newborn's first day of life did not identify microorganisms.

In a developmental appointment in Pediatrics, the mother revealed that she was very satisfied with the conduct of the team that provided her with life without serious sequelae and a healthy child. In the first year of life, the boy achieved all developmental milestones.

Nine months after the intervention, surgery records of the mother documented an incisional hernia, lacking plastic repair indication due to its large dimensions.

## 3. Discussion

More than a century has passed since *Edmund A. Babler* (1874-1930) declared *“the mortality of appendicitis complicating pregnancy and the puerperium is the mortality of delay”*. Even so, this statement remains valid nowadays, proven by several cases of a perforated appendix with increased maternal and fetal mortality [19].

In the extreme critical scenario, maternal sepsis is an uncommon complication of pregnancy demanding expeditious diagnosis, prompt identification of the source of infection, and targeted management [20].

The presence of a second dependent patient—the fetus in a life-threatening maternal condition may generate a conflicting obstetrical issue concerning induction of abortion in early pregnancy and timing of delivery in late pregnancy [17, 21].

For previable pregnancies, there is no clear evidence in favor of interrupting an early pregnancy to improve the mother's health [22]. Radical termination of pregnancy to execute an appendicectomy is not evidence-based, and optimizing maternal status is paramount [14, 17, 18]. Likewise, in advanced pregnancy, a concomitant cesarean is not recognized as a part of treatment for the surgical condition and should only be performed for obstetrical reasons. The use of tocolytics is controversial, as studies have failed to prove benefits with an increased risk of pulmonary lesion [23]. For a pregnant woman dealing with serious cardiovascular compromise due to sepsis, a delivery/induction of abortion could lead to higher maternal and fetal mortality, unless chorioamnionitis or septic abortion has occurred [24]. Except after 38 weeks, a c-section represents a transference of the maternal problems to the newborn, exposing it to the unnecessary risks of premature birth, like respiratory distress syndrome [17].

However, there are advocates of elective termination of pregnancy complicated by appendicitis, but this controversial subject is notably scarce in the literature. *Horntrich* et al. stated that *“caesarean section should be precedent to appendectomy, at least in cases of advanced appendicitis and when the foetus is viable”* [25]. *Ford* et al*.,* in a recent review, affirmed that in pregnancy before viability when the mother becomes ill termination of pregnancy is mandatory, preventing the septic status [26].

A few cases of induction of abortion following diagnosis of an acute abdomen in pregnancy can be found. *Cohen-Kerem* et al., in a systematic review, concluded that the rate of elective termination of pregnancy in a sequence of nonobstetrical surgery was 1.3% [27]. A paper included in this review conducted by *Tamir* et al. studied 84 pregnant women who underwent laparotomy after the diagnosis of acute appendicitis and determined 7 therapeutic abortions in that population [15]. Another paper integrated into the review that followed 44 pregnant patients with a diagnosis of biliary colic or cholecystitis stated that 3 patients interrupted their pregnancy during the first trimester because of persistent symptoms and the priority of an earlier cholecystectomy [28]. *Ueberrueck* et al. retrospectively analyzed 94 appendicectomies for suspected acute appendicitis during pregnancy and reported that the rate of therapeutic or requested abortion was 21.7% in the first trimester [29]. *Qihui* et al. investigated retrospectively 26 pregnant women with acute pancreatitis and declared that 9 cases requested termination of pregnancy and 5 cases were submitted to induction of abortion [30].

The rationale for the termination of pregnancy was not established in most papers. Some reasons mentioned are related to the concern with pregnancy gradually complicating the critical condition and to the fear of teratology as a result of medications administered, allegedly harmful to the fetus [30].

Our case report shows that conservative obstetrical behaviour carried out since the beginning, facing the threatening diagnosis of maternal shock septic, allowed the achievement of well-being for the mother and the fetus. Another feature highlighted in our case is the successful management of gestational abdominal compartment syndrome, whose evidence is practically absent in the literature. Few cases reported treatment of abdominal compartment syndrome in pregnancy. *Turnock* et al. described a case of acute perforated second-trimester appendicitis associated with intraabdominal sepsis and abdominal compartment syndrome. They applied a temporary abdominal closure with negative pressure wound therapy for nearly 1 week with excellent surgical and obstetrical results [31]. In our report, the abdomen was left open for 4 days despite the gravid uterus, and the cooperation between surgical and obstetrics teams, with intensive care support, made the complete restoration of maternal health possible, reaching the best obstetrical outcome.

A comparative table of previous studies on appendicitis in pregnancy is presented below (Table 1).

We also question whether the two sepsis clinical pictures (maternal and newborn) are related or whether they are independent. Considering that the maternal infectious condition resolved at the transition to second trimester, that the pregnancy continued uneventfully to term, and that 27 weeks separate the resolution of maternal and newborn sepsis, with no prebirth signs indicating intra-amniotic infection before the diagnosis of neonatal sepsis (no leukocytosis, maintenance of apyrexia during labor, absence of purulent vaginal discharge or pain on uterine palpation, absence of fetal/maternal tachycardia, intact membranes at the beginning of cesarean section, and artificial rupture of membranes intraoperatively with liquid clear amniotic), the hypothesis that they are two independent infectious situations seems to be the most likely.

As strengths of the study, the authors mention the fact that there was successful management in terms of several successive and rapidly deteriorating complex pathologies of the maternal clinical condition, such as acute appendicitis, generalized peritonitis, and abdominal compartment syndrome as a complication of laparotomy, aggravated and with mutual potential for atrocity by a fetus that was developing in this environment in the final stage of the first trimester. In addition, there was also an efficacious approach to emergent delivery and treatment of neonatal sepsis. All this effective management with the best maternal and perinatal outcome was based on the step-by-step coordination and communication of an in-hospital multidisciplinary team, consisting of obstetricians, general surgeons, intensivists, neonatologists, specialists in maternal-fetal medicine, and anaesthesiologists who joined knowledge and efforts in a common objective, the achievement of maternal stability with preservation of gestational and neonatal integrity. This article also emphasizes the importance of the low threshold of suspicion of appendicitis, even when the symptoms are confused with the normal ones of the pregnancy state or others that do not fit the usual clinical picture of acute appendicitis. In addition, it is a warning to raise awareness of the scarce literature on appendicitis in pregnancy, reinforcing the need for an active and critical discussion of these rare cases among the teams to promote further theoretical-practical knowledge, namely, about the management of the infectious pathology of appendicitis, peritonitis, sepsis, and abdominal compartment syndrome and how to manage the pregnancy itself in the limbo of these potentially fatal diagnoses.

As a limitation of this study, the authors cite the difficulty in diagnosing an acute abdominal process in pregnant women, either because of the anatomical changes that modify the appendicular location or because of the typical symptoms of pregnancy that camouflage a true pathology in aggravation. This obstacle explains the high rate of peritonitis in this population. Theoretically, emergent surgery should not be delayed in pregnant women as in nonpregnant women. However, the uncertainty of the diagnosis and the concern with fetal integrity during and after the surgical procedure generates a postponement in the decision of urgent intervention in the pregnant woman. Another potential limitation is related to the scarce literature regarding the characteristics, pathophysiology, epidemiology, risk factors, and management of appendicitis, peritonitis, and abdominal compartment syndrome in pregnancy, due to the very small number of inherent studies published, while those that exist have high variability in the diagnostic methods and surgical techniques used, potentially reflecting local practical experience and individual surgeon preference. And the emergency scenario itself dictates that not much time is wasted on audio-visual documentation that could help both “predict the unpredictable” and expedite practical approaches to similar cases in the future. A meta-analysis of all existing studies could serve to create a uniform protocol that facilitates clinical decisions in emergency medicine for pregnant women.

In conclusion, acute appendicitis is the main nonobstetrical cause for surgical intervention in pregnancy. A high index of suspicion, a low threshold to perform a life-saving operation, and the conservative obstetrical patterns carried out by surgical and obstetrics teams were the cornerstones for the improvement of mother's health, allowing her to proceed with the pregnancy and ultimately have a full-term newborn.

## Figures and Tables

**Table 1:** Comparative table of previous studies reporting appendicitis in pregnancy..

**Reference**	**Type of study**	**Pain location/other symtoms**	**Trimester**	**Management**	**Maternal outcomes**	**Fetal or neonatal outcomes**
Babler 1908 [19]	Case report	Sudden, progressive, severe right lower abdominal pain becoming excruciating associated with vomiting	Third (30 weeks)	Open surgery	Perforated appendix; large appendiceal abscess (discharged on postoperative D35); phlebitis in the left thigh (1 week after discharge)	Preterm labor (D2)
Tamir et al. 1990 [15]	Case series (84 patients; 54 patients with appendicitis pathologically confirmed)	Diffuse or periumbilical pain migrating to the right lower abdominal quadrant (48%); right lower quadrant pain only (28%); nausea/vomiting (91/81%); anorexia (70%); diarrhea (31%); constipation (4%)	First (32%); second (44%); third (16%)	Open surgery: right transverse muscle-spitting incision over the point of maximum tenderness (79%); low midline vertical incisions (13%); laparoscopic surgery: completed (1%), initially underwent diagnostic laparoscopy (4%) in the first trimester	Confirmed appendicitis: perforation rate: 43% (all with symptoms >24 h); periappendiceal or pelvic abscess: 22%; wound infection: 13% (11% perforated); no long-term maternal morbidity or mortality	Spontaneous abortion (2%); preterm labor (27%); failure of tocolysis (5%); Apgar score at 5 min <7 (5%); negative laparotomies: perinatal death (17%); extreme perinatal morbidity (3%)
Turnock et al. 2016 [31]	Case report	Progressive right/left lower and right upper quadrant abdominal pain associated with dysuria, nausea, and vomiting with oral intolerance in the previous 5 days	Second (15 weeks)	Laparoscopic approach with conversion to laparotomy (due to massive bowel distention and purulent ascites); temporary abdominal closure due to acute compartment syndrome with saline-dampened surgical towel placed over cassette cover; peritoneal toilet with inspection of the ileocolic anastomosis (D4); fascial closure (D6); delayed primary closure of the laparotomy incision (D12)	Ileocecum abscess; perforation of the appendiceal base with extension into the cecum; cecal necrosis (discharged on postoperative D15); no surgical complications at 5 years postdelivery	Term spontaneous vaginal delivery; child obtained all developmental milestones
Tase et al. 2017 [4]	Systematic review (43 articles)	Right lower quadrant pain (60-100%); nausea, vomiting, and anorexia common and indistinguishable from pregnancy related symptoms	First (30%); second (45%); third (25%)	Both open and laparoscopic surgery safe without statistically significant difference in perioperative obstetric or neonatal outcomes; no advisable medical management due little evidence on safety	Perforation rate: 20.3-43% (66% if delay in surgery >24 h; 8.7% first trimester, 12.5% second trimester, 26.1% third trimester)	Fetal loss: 1.5% delayed diagnosis, nonperforated; 35-55% delayed diagnosis, perforated appendix; delivery rate: 15-45% (preterm labor highest in the first week following surgery)
Hata et al. 2020 [32]	Case report	Acute epigastralgia, followed by right lower abdominal pain and vomiting	Third (27 weeks)	Laparoscopic surgery with reduced-port approach	None (discharged on postoperative D8)	Vaginal delivery at term
Tavakoli et al. 2020 [33]	Case report	Acute onset of sharp right abdominal pain associated with nausea and a single episode of vomiting	Third (37 weeks)	Conservative: intravenous antibiotics with complete resolution of abdominal pain; induction of labor (D3)	None (patient's pain and clinical status stable on D3 and discharged on postoperative D6 with a 10-day course of oral antibiotic; patient denied elective appendectomy at 20-months)	Uncomplicated vaginal delivery (D4)
Matsui et al. 2020 [34]	Case report	Diffuse abdominal pain migrating to the right lower abdominal quadrant started the day before admission	Second (20 weeks; dichorionic diamniotic twin pregnancy)	Laparoscopic surgery (3 trocars; insufflation pressure 10 mm Hg; left lateral tilt; ultrasonic energy)	None (discharged on postoperative D9)	Uncomplicated elective cesarean section (38 weeks)
Saleh et al. 2020 [35]	Case report	Persistent, severe, exacerbated by movement lower abdominal pain associated with loss of appetite in the previous 2 days	Second (17 weeks)	Laparoscopic surgery	Acute, nonperforated appendix; pelvic abscess; peritoneovaginal fistula (discharged on postoperative D5; endometriosis and decidualization on pathological examination of the appendix)	Uncomplicated spontaneous vaginal delivery (40 1/7 weeks)
Ghannouchi et al. 2021 [36]	Case report	Right iliac fossa pain in the previous 2 days	Third (32 weeks)	Planned appendectomy	None (discharged on postoperative D2; appendicular deciduosis on microscopical examination)	Uncomplicated delivery (39 weeks)
Sanders-Davis et al. 2021 [37]	Case report	Generalized abdominal pain migrating to right lumbar region associated with loss of appetite and vomiting; no respiratory symptoms (PCR SARS-CoV-2 infection positive)	Third (33 1/7 weeks)	Open surgery: right-sided transverse incision guided by the available imaging to allow access to the cranially displaced appendix	Perforated appendix with local peritonitis (worsening respiratory function after appendectomy with a diagnosis of bilateral pneumonia; discharged on postoperative D7)	Emergent cesarean section (33 6/7 weeks); neonatal respiratory distress syndrome with oxygen requirement at high pressures on mechanical ventilation, extubated at 24 hours of age (PCR SARS-CoV-2 negative at D3 and D5)

D: day.

## Data Availability

All data generated or analyzed during this study are included in this published article.
